# Polyphenol and metal ion-reinforced supermolecular hydrogels incorporating nanofiber drug and peptide for annulus fibrosus regeneration

**DOI:** 10.7150/thno.106913

**Published:** 2025-04-21

**Authors:** Long Xin, Xiaolin Li, Yang Yang, Pan Chen, Yi Li, Jianhua Liu, Kangbo Chen, Peipei Su, Shuaishuai Feng, Shiping He, Xinwei Xu, Wei Wang, Weixing Xu

**Affiliations:** 1 Orthopedics Laboratory, Department of Orthopedics, Tongde Hospital of Zhejiang Province, Hangzhou, 310012, China.; 2 Engineering Research Center of Functional Materials Intelligent Manufacturing of Zhejiang Province, ZJU-Hangzhou Global Scientific and Technological Innovation Center, Zhejiang University, Hangzhou, 311215, China.; 3 College of Chemical and Biological Engineering, Zhejiang University, Hangzhou, 310027, China.; 4 Zhuji Affiliated Hospital of Wenzhou Medical University, Zhuji, 311899, China.; 5 The Second Affiliated Hospital of Zhejiang University, Hangzhou, 310009, China.; 6 College of Mechanical and Automotive Engineering, Zhejiang University of Water Resources and Electric Power, Hangzhou, 310018, China.; 7 Department of Clinical Laboratory, Shenzhen Second People's Hospital, Shenzhen, 518035, China.

**Keywords:** KGN@TA nanofiber, ROS scavenge, anti-inflammation, MSC recruitment, annulus fibrosus regeneration

## Abstract

**Rationale:** Following the structural destruction of annulus fibrosus (AF), the early-stage damage manifests as symptoms such as an inflammatory phenotype and loss of mechanical support. The microenvironmental deterioration at the injury site, the limited population, and the inadequate differentiation of intrinsic stem/progenitor cells impede the efficient repair of AF. To address the aforementioned challenges, we developed a dual-drug-loaded hydrogel system to achieve systematic and functional annulus fibrosus tissue repair.

**Methods:** A tannic acid-crosslinked gelatin-based hydrogel scaffold with the addition of Mn^2+^ was designed to work as a platform to provide mechanical support, antioxidant capacity, and immune-modulating function. The kartogenin-loaded nanofiber and SDF-1α mimic peptide were also incorporated into the hydrogel system to facilitate the recruitment of endogenous stem cells and direct AF tissue regeneration.

**Results:** The resulting hydrogel scaffolds exhibit excellent biogenic properties while achieving mechanical properties similar to those of AF. The composite scaffold also enhances ROS clearance and promotes M2 polarization of macrophages to improve the inflammatory microenvironment during early-stage injury. Furthermore, the sustained release of kartogenin-loaded nanofiber and SDF-1α mimic peptide effectively enhances endogenous stem cell recruitment, promotes cartilage differentiation, and facilitates specific extracellular matrix deposition, thus meeting requirements for late-stage AF repair.

**Conclusion:** The findings demonstrate the potential of a multifunctional, high-strength supramolecular hydrogel loaded with dual drugs for the functional regeneration of AF tissue.

## Introduction

Intervertebral disc degeneration (IVDD) caused by disruption of the annulus fibrosus (AF) is a leading cause of low back pain, which severely affects the quality of life 1, 2. Due to their poor self-repairing nature, a mature regeneration of AF tissue has not been achieved effectively once damage occurs 3, 4. The construction of native annulus integrity and subsequent restoration of the intervertebral disc (IVD) function are crucial for alleviating disc degeneration 5. Previous studies primarily focused on biomedical tissue engineering scaffolds but often neglected the need for endogenous AF healing and the advancement of stem cell homing and cellular differentiation strategies for the supplementation of AF lesions 6. Thus, there is an urgent need to develop effective AF repair techniques for long-term regeneration and to effectively restore native tissue structure and function.

The key to achieving high-quality repair lies in constructing an AF scaffold that mimics the hierarchical structure of native tissue. The destruction of the AF structure results in early-stage damage, leading to the loss of local physical support function and triggering an inflammatory response at the injured site. Therefore, biomaterials capable of rapidly filling and replacing the defect site, thereby restoring the supportive function of the AF, have garnered significant attention. TA (Tannic acid)-based composite systems have been developed for biomedical applications, such as high-strength polyphenol composite hydrogels with various types of ligand molecules, including drug molecules, functional metal ions, and proteins. These systems have demonstrated excellent mechanical properties and hold promise for biomedical application properties in holding promise for biomedical applications 7, 8. Dong et al. developed a multifunctional TA-mediated multifunctional hydrogel that facilitated enriched mechanical properties and promoted osteogenic and chondrogenic differentiation of stem cells. This innovative approach has garnered significant attention for its potential to repair osteochondral defects in the treatment of osteoarthritis. 9, 10. Notably, incorporating metal-ligand interactions represents a prominent approach for integrating simulated factors into scaffolds to form nanoparticulate carriers and hydrogels while also imparting biochemical properties such as anti-oxidation and anti-inflammation 11, 12. Therefore, the TA/Mn^2+^ system is anticipated to effectively address both the early injury-induced physical support performance mismatch and rectify the inflammatory microenvironment. Also, by optimizing metal ions and their ligands, self-polymerization of the TA/Mn^2+^ complex endows a versatile multifunctional platform through an in-situ metal-ion-assisted deposit approach.

Numerous techniques have been developed to employ biomaterial-based approaches for AF repair and regeneration at a later stage, such as cell delivery and recruitment approaches, and bio-factor delivery to enhance tissue healing 13. Unfortunately, the clinical application of AF tissue engineering has been impeded due to the intricate and multi-step manufacturing processes involved in existing preparation methods. So far, SDF-1α, known as thymus-expressed chemokine, has demonstrated a remarkable capacity for recruiting stem cell-like cells 6, 14, 15. They possess an excellent ability to induce cell migration and initiate recruitment of MSCs from the intact surrounding tissue into the annulus defect area, thereby promoting tissue regeneration 16.

With the prolongation of injury duration, the augmented degradation metabolism of ECM structure at the injured site impedes tissue regeneration, thereby emphasizing the significance of directing stem cell differentiation and stimulating ECM anabolism. Kartogenin (KGN) has been widely used for tissue repair and regeneration, including cartilage regeneration 17, 18, disc repair 19, and tendon-bone healing 20. The study conducted by Yu et al. demonstrated that TA enables the formation of a hydrogel capable of loading KGN, which effectively promotes MSC differentiation, chondrocyte proliferation, promoting anabolism, and suppressing chondrocyte catabolism under IL-1β-induced inflammation 19.

**Figure 1:**
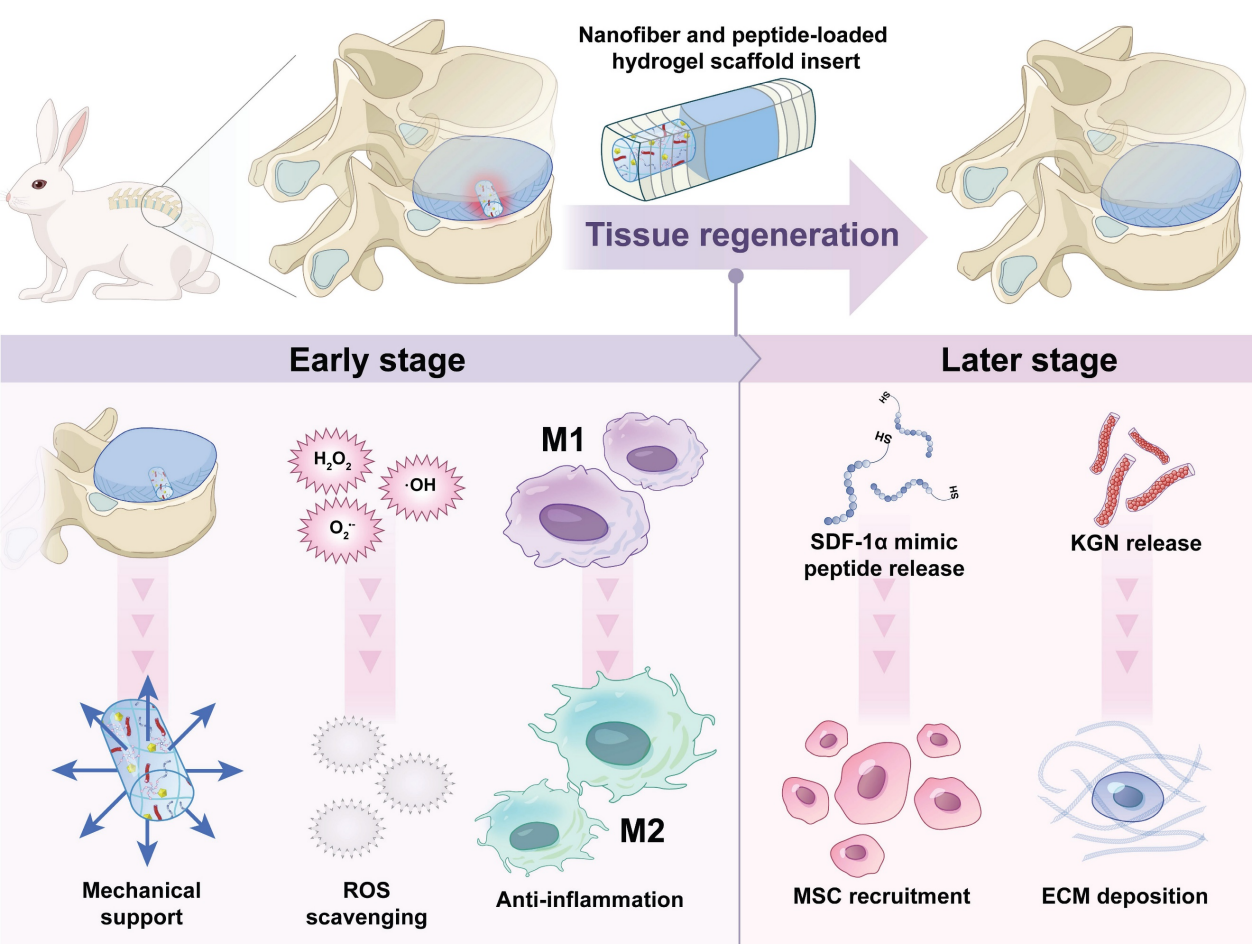
Schematic illustration of the components in nanofiber and peptide-loaded hydrogel scaffold for annulus fibrosus regeneration.

To date, no study has reported the assessment of the system of TA/Mn^2+^ based hydrogels loaded with KGN-loaded TA nanofiber (KGN@TA) and SDF-1α mimic peptide for annulus tissue engineering. Here, a controlled-release TA/Mn^2+^ system loaded with dual drugs was developed. The composite hydrogels, loaded with SDF-1α and KGN nanofiber, could enhance the recruitment, migration, and chondrogenic differentiation of endogenous stem cells, promote mechanical support, reduce inflammation, mitigate oxidative stress, and foster tissue regeneration. Simultaneously, these composite hydrogels may alleviate the inhibition of anabolism in normal chondrocytes within the inflammatory microenvironment following AF injury, thereby promoting AF regeneration, as illustrated in Figure 1.

## Methods

### Preparation and characterization of drug-loaded nanofibers

KGN dissolved in ethanol was added to 5 mL of distilled water dropwise and stirred at 900 rpm overnight to evaporate the organic phase. Then, 5 mL TA with a concentration of 10 mM was added to the KGN suspension solution, and the pH was adjusted to 9. After constant stirring for 12 h, the TA-coated nanoparticles were collected with a centrifuge and washed with distilled water 3 times. The nanofibers were prepared by passing the nanoparticles through a filter with a pore size of 0.45 μm.

The surface morphology of collected nanofibers was examined by transmission electron microscope (TEM) (HT-7700, Hitachi, Japan), scanning electron microscopy (SEM) (Sigma 300, ZEISS, UK), and scanning probe microscope (Multimode, VEECO, USA), following the established protocol 21. Surface atomic composition was analyzed by a multifunctional nano infrared spectrometer (Nano IR) (nanoIR2-fs, Anasys Instruments, USA).

### TA/KGN@TA/SDF-1α/Mn^2+^/Gel hydrogels synthesis and mechanical properties

The hydrogels were prepared by mixing gelatin, TA solution, and with or without MnCl_2_, KGN-loaded TA nanofiber, and SDF-1α mimic peptide under constant stirring within a water bath at 50 °C. The final concentration within the hydrogel scaffold would be 15% gelatin (w/v), 20 mg or 40 mg/mL TA, with or without 10 mM MnCl_2_, 10 mg/mL KGN@TA nanofiber, and 10 mg/mL SDF-1α mimic peptide. Hydrogel mixtures were filled into molds and set at 4 °C overnight before use. The hydrogels were freeze-dried for 24 h and followed by sectioning into thin layers before being observed by SEM.

The mechanical properties of the hydrogels (prepared according to Table S1) were characterized with universal testing machines (Instron 2344 Microtester) at room temperature. Cylindrical samples (diameter 12 mm, height 10 mm) were prepared for compressive properties while dumbbell samples (2 mm in width, 10 mm in gauge length) were prepared for tensile tests. The compression and extension rates were fixed at 5 mm/min, and compressive strength was conducted at a strain of 90%. At least six samples were used in each test.

The rheological characteristics of hydrogels were evaluated using a parallel-plate geometry rheometer (Discovery HR-2 Rheometer, TA Instruments). Cylindrical samples with a diameter of 20 mm and 5 mm in height were subjected to standardized testing procedures. In brief, frequency sweep tests were conducted under a constant strain of 1% at 37 °C, with frequencies ranging from 0.1 to 100 rad/s. The shear strain was switched from strain = 1% to strain = 500% and was repeated three times with an interval of 50 s. At least three samples were tested for each hydrogel group.

### Hydrogel degradation and *in vitro* release properties

The degradation behaviors of hydrogels were conducted with cylinder samples (2 mm in diameter and 4 mm in height). The initial weight of all samples was recorded as W_0_ and then transferred into trans-well inserts. Each sample was incubated in 2 mL PBS with Collagenase type II (2 μg/mL) at 37 °C in a shaker (100 rpm). Essentially, the buffer was completely removed from the wells, and the weight of hydrogels was measured as W_t_ at the set time points. The remaining gel mass of hydrogel samples can be calculated as (W_t_/W_0_) × 100%. At least three samples were tested for each hydrogel group.

The release of KGN and manganese ions in hydrogels was conducted. The hydrogels were soaked in PBS with Collagenase type II (2 μg/mL) and incubated at 37 °C with continuous rotation (100 rpm). The buffer samples were collected at each time point, with 1 mL being withdrawn and replaced with an equal volume of fresh buffer. Subsequently, the quantification of KGN in PBS was performed using high-performance liquid chromatography (HPLC) (Agilent 1100 HPLC System; Agilent Technologies, USA). Specifically, the ion concentration of the supernatants at decimated time points was determined by an inductively coupled plasma optical emission spectrometer (ICP-OES, Avio 200). At least three samples were tested for each group.

### *In vitro* antioxidant activity

The antioxidant activity of these hydrogels was evaluated *in vitro* using the DPPH, ABTS, and PTIO radical scavenging assay. Each assay was conducted with three replications. Briefly, the DPPH detection reagent (0.1 mM) was prepared with methanol. Subsequently, the hydrogel samples (20 mg) were immersed in 5 mL of the DPPH solution and incubated at 37 °C in darkness. The absorbance of the DPPH solution at 517 nm was measured using a UV-Vis spectro-photometer at the set time interval.

The ABTS stock solution was prepared by combining 7.4 mM of ABTS with 2.6 mM potassium persulfate in 95% Ethanol and stored in the dark at room temperature for approximately 12 h before use. The stock solution was diluted 2 times before experimenting and was conducted following the abovementioned method. The percentage of ABTS scavenging was determined based on absorbance at 734 nm.

For PTIO inhibition experiments, the PTIO reagent was prepared at a concentration of 0.2 mg/mL with 95% Ethanol. The experiment was carried out following the methodology described above. The percentage of PTIO scavenging was determined based on absorbance at 560 nm. Measurements were subsequently conducted in triplicate, and the antioxidant efficacy was determined using the following formula:

Antioxidant efficiency (%) = (A_Control_ -A_Sample_) / A_Control_ × 100%

The absorbances of the working solution with or without hydrogel samples at a specific interval were denoted as A_Sample_ and A_Control_, respectively.

### *In vitro* characterizations of macrophage polarization

For the macrophage polarization assay, the macrophage cell line, Raw 264.7 cells, was seeded in the lower chambers of sterile 24-well trans-well plates at a density of 1 × 10^6^ cells per well. The control group was conducted without any treatment, while cells in the lipopolysaccharide (LPS) and LPS+GT4MKS group were cultured with LPS (100 ng/mL)-loaded complete medium for 24 h. The GT4MKS hydrogel was then loaded in the upper chambers of the LPS + GT4MKS group and inserted after the fresh complete medium was replaced. The cells were further treated for another 24 h before being harvested and used in subsequent experiments. Immunofluorescent staining was performed to analyze the expression of cell markers for M2 and M1. The cell samples were fixed in 4% paraformaldehyde, and incubated with rabbit anti-CD206 (1:500, Proteintech, USA), rabbit anti-iNOS (1:1000, Proteintech, USA), and rabbit anti-CD86 (1:500, Proteintech, USA) primary antibodies followed by incubation with goat anti-rabbit-Alexa Fluor-594 (1:1000, eBioscience, USA) secondary antibodies and DAPI (10 μg/mL). Fluorescence imaging was performed with a Laser Scanning Confocal Microscopy (AX-SHR, Nikon, Japan).

### Cell migration test

The cell migration assay was performed with BMSCs, which were isolated from 5 male SD rats (6-8 weeks old). In brief, the hydrogels were rinsed with PBS and air-dried before use. The BMSC cells were seeded in a cell culture plate (1×10^5^ cells per mL), and a scratch was created using a 200 μL pipette tip when 80% confluence was reached. After being washed with PBS 3 times, the cells were incubated with hydrogel extractions, and photos of the wound area were taken at 0 h, 12 h, and 24 h, respectively. The wound area was analyzed by ImageJ software (version 1.8.0, NIH), and the wound closure rate was calculated by the formula with 3 replications:

Closed rate (%) = (A_0_-A_t_)/A_0_ × 100%

Where A_0_ represents the wound area at 0 h and A_t_ is the wound area at a predetermined time.

Another cell migration assay was investigated with trans-well plates. The hydrogels were placed in the lower chamber of the 24-well trans-well plate, while 1×10^5^ cells per mL BMSCs were seeded into the upper chamber. Each chamber's cell number and viability were recorded and summarized. 3 replications were performed for each group.

### Chondrogenic differentiation

The rat BMSCs were seeded in the lower chambers of 24-well trans-well plates (1×10^7^ cells per mL). The control group was left untreated, while hydrogels were loaded in the upper chamber. The fresh medium was replaced every other day, and the KGN-treated group was replaced with a complete medium with the addition of KGN (50 μM). Cells were harvested at 1 week and 3 weeks with or without treatment, and each group was performed with 3 replicates.

### qRT-PCR assessment

The RT-qPCR assessment was applied to investigate the gene expression in cells or tissues. For the cell samples, 1 mL TRIzol buffer was applied to each well of the 6-well plate after discarding the cell culture medium. The AF tissue was preserved in liquid nitrogen and homogenized before being resuspended in 1 mL of lysis buffer (50 mM Tris-HCl/0.1% Triton X-100/2 mM EDTA/100 mM NaCl/1 mM protease inhibitor) at 4 °C for 30 min.

The total RNA of cells and tissues was extracted from the specimens using the TRIzols Plus RNA Purification Kit (Invitrogen) and RNase-Free DNase Set (Qiagen). Approximately 1 μg of extracted RNA was then reverse transcribed into cDNA. The real-time PCR was performed on a CFX384 real-time PCR system (Bio-Rad) using Power SYBR Green PCR Master Mix (Applied Biosystems), using specific primers listed in Table S2, and results were normalized to GAPDH and quantified using the 2^-ΔΔCt^ method.

### Surgical procedures

All procedures were approved by the Institutional Animal Care and Use Committee of the Zhejiang Center of Laboratory Animals (Permit No. ZJCLA-IACUC-20030108) and followed the National Research Council's Guide for Care and Use of Laboratory Animals.

A total of 30 New Zealand rabbits (~2.50 kg) were used in this work. The rabbits were randomly assigned to either the 4-week or 12-week survival groups. The surgical procedure was previously described, which involved a retroperitoneal approach to expose consecutive levels of rabbit IVD, specifically L3/4, L4/5, and L5/6 5, 22. Annular cylindrical defects (diameter: 2.0 mm, depth: 4.0 mm) were created on the anterolateral disc and then implanted randomly with or without hydrogel scaffolds. Six groups were established, including the control group receiving no treatment, the Blank group receiving an annular defect without implantation, the Geln/TA (GT) implantation group, the Geln/TA/Mn^2+^ (GTM) implantation group, the Geln/TA-KGN/Mn^2+^ (GTMK) implantation group, and the Geln/TA-KGN/Mn^2+^/SDF-1 (GTMKS) implantation group. Following surgery, the rabbits were permitted individually free cage activity and were sacrificed at 4 and 12 weeks post-operation.

### Magnetic resonance imaging (MRI) and Disc height assessment evaluation

The *in vivo* MRI of the lumbar spine was conducted utilizing a 3.0-T MRI system (Signa, General Electric, Milwaukee, Wisconsin). The MRI scanner parameters were set according to the previously described protocol, and the structure of NP was evaluated using T2-weighted sections in a sagittal plane at 4 and 12 weeks after post-operation. Finally, the alteration of NP was assessed based on Pfirrmann's classification scores 23. The rabbits were put in the prone position, and sagittal X-ray fluoroscopy images were acquired at 4 and 12 weeks intervals post-surgery (n=6 per time point). DHI of the experimental disc was calculated and normalized to the native healthy disc, following previously described methods 22.

### Histological and immunohistochemistry evaluation

The rabbits were euthanized at the designated time points, and the samples were fixed in 4% paraformaldehyde, decalcified in 10% ethylenediaminetetraacetic acid (EDTA), and embedded in sections. H&E or Safranin-O/fast green staining was conducted for histological evaluation. Histological staining images were captured using an optical microscope (IX70, Olympus). Images were adjusted and analyzed using ImageJ software. Histological grading of disc degeneration was compared to the control group based on the established evaluation scale 24, 25.

Immunofluorescence staining was performed after antigen retrieval. The sections were treated with 3% H_2_O_2_ for 15 min and 10% goat serum for 15 min. The sections were then blocked with 5% BSA solution, incubated with 1:100 diluted IL-1β (ab9722, Abcam), IL-6, TNF-α (KF782-1-1 and 33648, Nanjing Jiancheng Bioengineering), CD-90, CD105 (AF300369 and AF20160, AiFang biological), iNOS and CD-206 (13120S and 24595S, CST) primary antibodies for 12 h at 4 °C. Subsequently, the sections were incubated with 1:200 diluted Alexa-fluor 647 labeled secondary antibodies (Ab150075, Abcam) for 1h at room temperature. DAPI staining was employed to visualize the cell nucleus. Immunofluorescence staining images were acquired using an automatic digital slicing scanner (VS120, Olympus) and analyzed by OlyVIA software (version 3.1, Olympus).

### Statistical analysis

All the quantitative data were presented as mean ± standard deviations. For cases with equal variances, distinct groups were compared using Kruskal-Wallis one-way analysis of variance and post hoc Mann-Whitney U-test. Statistical significance was considered when the p-value was less than 0.05.

## Results and Discussion

### Fabrication and characteristics of KGN@TA nanofiber

The constant release of biological factors and/or bio-activators plays a crucial role in tissue regeneration. TA, a naturally derived material extracted from food, has been employed for biomaterial coating due to its inherent ability to self-assemble in an alkaline environment 22. The TA coating offers a straightforward solution to the challenge of hydrophobic drug delivery.

**Figure 2:**
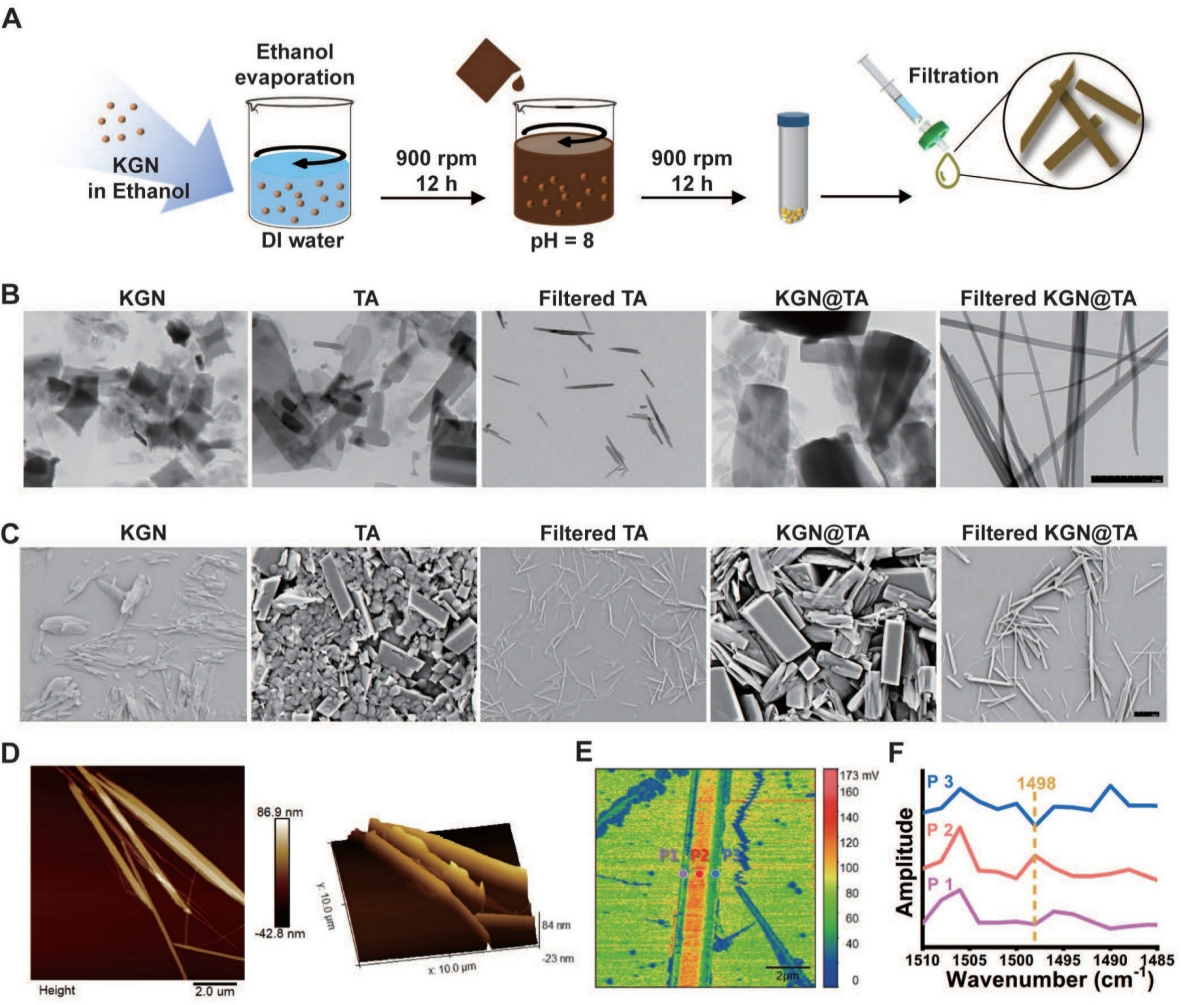
Fabrication and characterization of KGN-loaded TA nanofiber. (A) KGN@TA nanofiber preparation step. Representative TEM (B) and SEM (C) images of KGN particles, TA particles, TA nanofibers, and KGN@TA nanofibers (scale bar = 2 μm). (D) 2D and 3D AFM image of KGN@TA nanofibers. (E) Nano-FTIR spectroscopic mapping across a KGN@TA nanofiber and (F) absorption spectra of representative points.

The abounded phenolic hydroxyl groups present in TA facilitate the formation of a quinone outer layer on hydrophobic particles. In this study, we dispersed KGN solution in deionized water to generate insoluble KGN particles and followed with constant stirring in alkaline TA solution to achieve TA-coated KGN particles. With a simple fabrication process, hydrophobic KGN can be properly delivered, which offers a novel drug delivery method for hydrophobic compounds. Interestingly, upon further extrusion through a 0.45 μm filter, the particles underwent a transformation into fibers (KGN@TA nanofibers) (Figure 2A).

The unloaded TA particles also exhibited nanofiber formation; however, they were significantly smaller and shorter compared to the KGN@TA nanofibers (Figure 2B and 2C). Subsequent atomic force microscope (AFM) characterization of the KGN@TA nanofibers revealed their elongated spindle-shaped morphology, which was attributed to the self-assembly of smaller fibers (Figure 2D). Additionally, X-ray diffraction (XRD) analysis confirmed the successful formation of the TA complex with an evident peak at approximately 28°, while encapsulation of KGN within KGN@TA nanofibers resulted in a reduced density of this signature peak (Figure S1).

The structural formation of nanofibers was verified using Fourier transform infrared spectroscopy (FTIR), as shown in Figure S2. The wide peaks observed at 2600-3700 cm^-1^ in all cases were attributed to the presence of phenolic hydroxyl groups from TA within the nanofibers. The peaks at 1490, 1470, and 1432 cm^-1^ indicated the formation of a quinone structure through TA self-assembly, while the signature peak at 1530 cm^-1^ suggested the incorporation of KGN 23. Nano-FTIR analysis revealed a distinct difference in spectral power distribution between the outer layer and interior regions, confirming the core-shell architecture of KGN@TA nanofibers (Figure 2E). Furthermore, examination of absorption spectra from representative points confirmed variations between the outer layers and internal material within KGN@TA nanofibers (Figure 2F). The KGN encapsulation efficiency was also evaluated using KGN@TA nanoparticles and nanofibers, with only a slightly lower KGN loading observed in the nanofiber groups (Figure S3). These findings suggest that the TA-coated nanofiber has the potential to serve as an effective delivery system for hydrophobic drugs.

### Hydrogel preparation and characterization

At the early stage of injury, the absence of physical structure in the annulus fibrosus continues to undergo restoration and replenishment, thereby reinstating its inherent physical properties. With the rapid development of tissue engineering, AF tissue-like TA/Geln composite has been employed for achieving AF reconstructions in recent years. However, its application in AF repair remains at the experimental stage and has not been widely used in clinical practice. In this study, we established a Geln/TA/Mn^2+^ (GTM) complex that endows a versatile multifunctional platform with enhanced biomechanical and structural properties of regenerative tissue.

**Figure 3:**
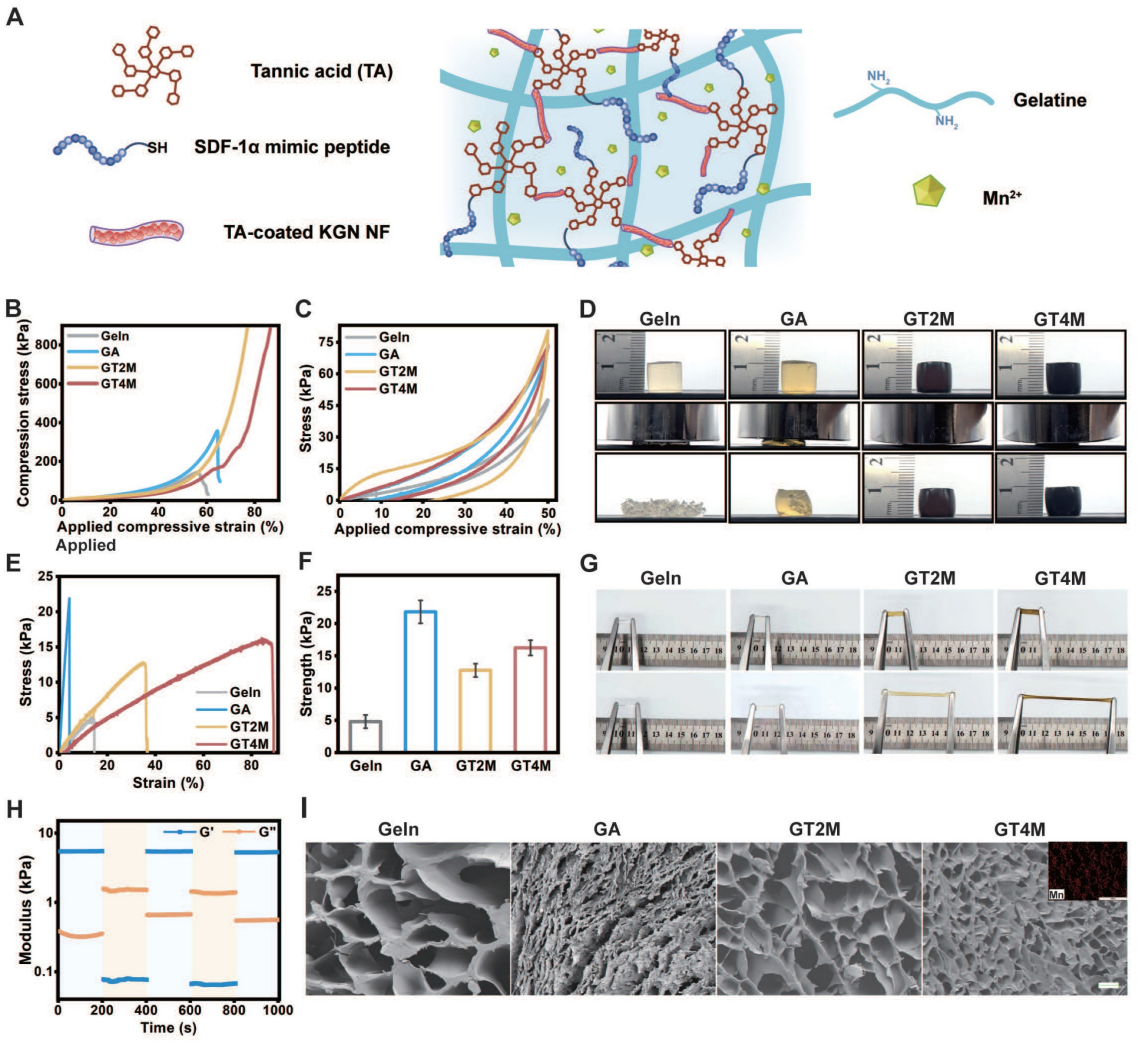
Mechanical evaluation of gelatin, glutaraldehyde-crosslinked gelatin, GT2M, and GT4M hydrogels (A). Representative compressive strain-stress curves (B), load and unload compressive curves (C), and compressive image of different hydrogels (D). Representative stretch strain-stress curves (E), the summary of stretch modulus (F), and (G) stretch image of different hydrogels. (H) Rheological properties of GT4M hydrogel under different strain. (I) The inner section of different hydrogels and manganese ion distribution in GT4M hydrogel (inserted Figure), scale bar = 100 μm.

The GTM hydrogel was prepared by a one-step cross-linking interaction between the catechol group of TA and the amino group of gelatin, followed by the addition of manganese ions (Figure 3A). The incorporation of metal ions like Mn^2+^ into the TA-crosslinked gelatin network significantly enhanced its mechanical strength, toughness, and flexibility 24. Hydrogels with varying component concentrations were prepared to investigate the mechanical properties of these materials (Figure S4). As demonstrated in Figure 3B, the GT2M (GTM with 20 mg/mL TA) and GT4M (GTM with 40 mg/mL TA) hydrogel exhibited remarkable strength and toughness.

Compressive tests confirmed a maximum compressive strength of over 900 kPa at 70% and 80% strain. In comparison, the pure gelatin only displayed a compressive strength of 150 kPa at a strain of 55%, which improved to 350 kPa at a strain of 65% when crosslinked with the commonly used crosslinker glutaraldehyde. The antifragility and recoverability of the hydrogel were evaluated through a loading-unloading cyclic compression test. As illustrated in Figure 3C, the hysteresis loop observed in GT4M indicates great energy dissipation at 50% strain. Notably, both GT2M and GT4M hydrogels fully recovered their original height after 10 consecutive loading-unloading cycles at 80% strain (Figure 3D), while pure gelatin and glutaraldehyde crosslinked groups broke after the first cycle. The almost overlapping hysteresis loops of each cycle suggest no breakage or permanent damage occurred in either GT2M or GT4M hydrogels. The tensile strength of the hydrogels was also evaluated, with the GT4M hydrogel exhibiting a higher tensile strength of 16 kPa at a strain of 90%, while the pure gelatin only reached 5 kPa at 16% strain (Figure 3E). Furthermore, summarized data confirmed that the glutaraldehyde crosslinked group displayed improved breaking strength; however, it fractured at a strain of only 6% (Figure 3F). Illustrations of the stretched hydrogels clearly demonstrate the superior elongation capacity of the TA and metal crosslinked hydrogel system (Figure 3G). The excellent mechanical properties observed in GT2M and GT4M hydrogels can be attributed to the hydrogen bonds formed between amine groups on gelatin and catechol groups, as well as to the strong Mn^2+^ chelating ability and self-assembly capability of catechol or galloyl groups on TA 25. The result was also confirmed with FTIR spectra of hydrogels (Figure S5). Consequently, an increased concentration of TA leads to enhanced mechanical properties. It is also confirmed that the combination of KGN@TA nanofiber and thiol end group modified SDF-1α mimic peptide only has a slight effect on the compression (Figure S6A) and stretch (Figure S6B) properties of the hydrogel system.

The rheological properties of the hydrogels were measured with oscillatory time scans, with low (strain = 1%) and high (strain = 500%) oscillatory strains applied repeatedly every 200 s. A lower loss modulus (G'') compared to the storage modulus (G') under high strain indicates the liquid-like characteristics of the GT4M hydrogel, while the return to original values under low strain after multiple cycles suggests the excellent self-healing ability of the system (Figure 3H). Further investigation confirmed that the freshly cut GT4M hydrogels were found to merge seamlessly after 30 s contact and remained intact under sustained stretching (Figure S7). Additionally, the hydrogel exhibited the ability to be reshaped into various forms at 37 °C (Figure S8). The rapid self-healing behavior of the hydrogels can be attributed to the formation of intensive hydrogen bonds among functional groups within the polyphenol/metal system, resulting in a relatively homogeneous distribution upon incision healing.

The porous structure of the hydrogel sample was examined using scanning electron microscopy (SEM) (Figure 3I). The non-crosslinked gel exhibits a pore size ranging from 210 μm to 300 μm, whereas the GT2M and GT4M groups obtained pore size distributions of 80 - 130 μm and 10 - 90 μm, respectively. It is reasonable to observe that crosslinked hydrogels possess a smaller pore size compared to pure gelatin samples due to higher TA concentration, leading to a denser system with reduced pore size. Furthermore, the evenly distributed Mn^2+^ contributed significantly to achieving superior mechanical strength through increased cross-linking density.

As demonstrated in Figure S9, the GT4M hydrogel displayed excellent adhesion properties to a variety of materials, including wood, polypropylene (PP), glass, metal, pork skin, heart, lung, and liver tissue. The lap shear test of GT4M with wood, PP, glass, metal, and pork skin was also conducted (Figure S10). The attachment of the hydrogel to the finger with or without latex gloves could adapt to the different degrees of finger bending. The strong adhesion of GT4M hydrogels can be attributed to the interaction between numerous active groups on the surface of skin tissue and hydroxyl groups present in the hydrogel matrix. In addition, the benzene ring in the hydrogel also engages in cation-π and π-π stacking with metal ions and tissues. At the same time, electrostatic and hydrogen bonding occur between the hydrogel and tissue surface all contributing to the adhesion ability of hydrogels 26. The adhesion of hydrogels to tissues is crucial to medical applications as it enables direct application to injured areas without the assistance of additional treatment.

The Mn^2+^-enriched TA-crosslinked hydrogel structure in this study demonstrates excellent mechanical properties, including antifragility, rapid recoverability, self-healing ability, elongation capacity, and strong adhesion properties. These characteristics are highly desirable materials for the regeneration of load-bearing AF tissue.

### ROS scavenging activity of hydrogels

ROS production and nonspecific inflammation generated during the pathological process of IVDD have been demonstrated to lead to the loss of transplanted stem cells 27, 28. Subsequent IVDD development, an unfavorable microenvironment characterized by oxidative stress, the release of inflammatory factors, and matrix degradation, leads to excessive cellular autophagy, apoptosis, and necrosis of stem/progenitor cells, thereby limiting the efficiency of AF repair 6. Therefore, the clearance of ROS could serve as a viable strategy for attenuating the inflammatory response in the injured area.

Polyphenols such as TA are widely acknowledged as natural antioxidants capable of reducing inflammation, prompting an investigation into the catalytic activities of the TA-crosslinked hydrogels with various substrates 29. It is also confirmed by previous studies that Mn^2+^ displayed excellent antioxidant activity and anti-apoptotic effects in the rat IVDD model 30.

**Figure 4:**
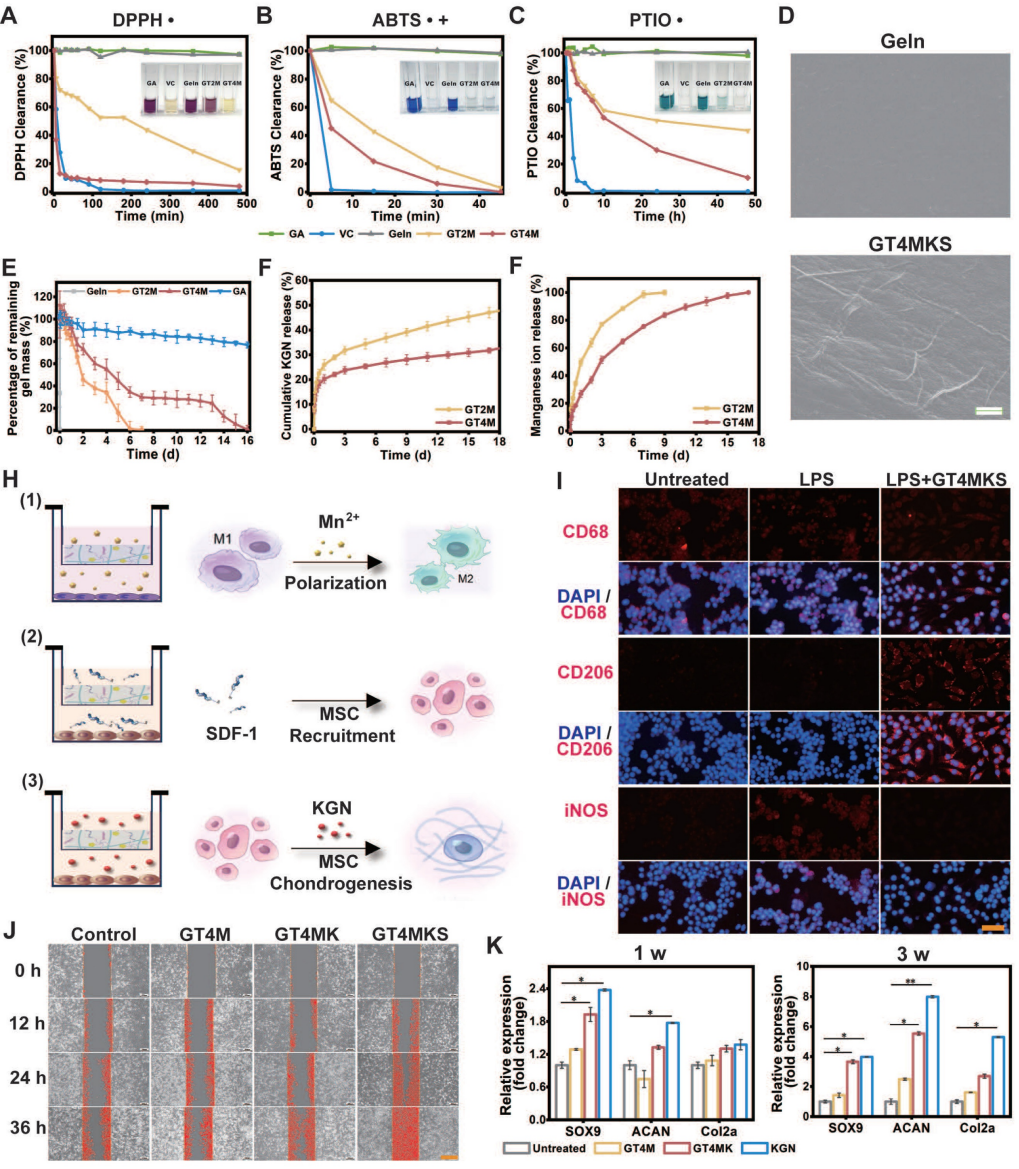
*In vitro* biological abilities of hydrogels. (A-C) ROS clearance of different hydrogels with DPPH, PTIO, and ABTS reagent. (D), Amplified SEM image of Gelatin and KGN@TA nanofiber-loaded hydrogel (scale bar = 1 μm). (E) *In vitro* hydrogel degradation in an enzyme environment. KGN (F) and manganese ion (G) are released from GT2M and GT4M hydrogel (n=3). (H) Schematic image of M2 polarization of macrophage, recruitment of BMSC, and chondrogenesis of BMSC while co-cultured with GT4MKS hydrogel. (I) Fluorescent staining of biomarkers (CD68, CD206, and iNOS) and DAPI in macrophages with or without LPS, LPS, and GT4MKS treatment (scale bar = 40 μm). (J) BMSCs migration with or without GT4M, GT4MK, and GT4MKS treatment (scale bar = 400 μm). (K) Gene expression of SOX9, ACAN, Col2a with or without GT4M, GT4MK, and KGN treatment in BMSCs (n=3).

Remarkably, the Geln/TA/Mn^2+^ complex demonstrated a substantial enhancement in antioxidant activity, resulting in oxidation rates approaching 90% for 2,2-diphenyl-1-picryl hydroxyl (DPPH) and 2,2'-Azinobis-(3-ethylbenzthiazoline-6-sulphonate) (ABTS) within 30 min and 2-phenyl-4,4,5,5-tetramethylimidazoline-1-oxyl 3-oxide (PTIO) within 48 h (Figure 4A, 4B, and 4C). The results confirmed that the TA/Mn^2+^ complex endows the scaffold with an anti-ROS function, which may lead to the protection of healthy cells by regulating the microenvironment after AF injury.

### *In vitro* biological abilities of hydrogels

Studies have shown that endogenous stem/progenitor cells play an essential role in AF regeneration, especially in the later stage of AF regeneration. One contributing factor to the transformation of regenerative AF tissue into fibrocartilage is the insufficient recruitment of stem cells within the damaged region 12. Therefore, it is imperative to consider the migration and retention, as well as proper differentiation, of endogenous stem cells in the injured area.

The biocompatibility of hydrogels was evaluated using both BMSCs and annulus fibrosus-derived cells (AFCs) before conducting further investigation. The hydrogels demonstrated minimal impact on cell viability, as evidenced by the maintenance of high levels of viability in both cell types. Specifically, BMSCs recorded viability above 100%, while AFCs maintained viability above 90% after being incubated in hydrogel extracts for a duration of 3 days (Figure S11).

The loading of KGN@TA nanofibers was confirmed by SEM, demonstrating their presence within the hydrogel structure (Figure 4D). TA and Mn^2+^ crosslinked hydrogel exhibited a prolonged degradation for 7 and 16 days in the GT2M and GT4M group at 37 °C with the existence of collagenase type II, compared to the non-crosslinked group, which degraded within 3 h (Figure 4E). The release tests for KGN were also consistent with the hydrogel degradation rate. The KGN loaded in the nanofiber demonstrated controlled release over a period of 16 days, while higher concentrations of TA resulted in an even slower release from crosslinked hydrogels (Figure 4F). At the same time, the Mn^2+^ also exhibited a similar release pattern, indicating that crosslinked hydrogels could prevent burst release of loaded components, with higher levels of TA contributing to enhanced controlled release ability (Figure 4G). Further experiments were conducted using the trans-well system to confirm the controlled release ability of hydrogels in modulating cell functions (Figure 4H).

The inflammatory microenvironment usually triggers an upregulation in extracellular matrix degradation metabolism at the site of injury, which hampers tissue repair 31. Therefore, the regulation of the local inflammatory microenvironment plays an essential role in AF restoration 32. It was confirmed in previous studies that TA/Metal ions (like Mn^2+^) could mediate the local inflammatory microenvironment 33. Macrophages, especially M2 phenotype macrophages, are critical factors in anti-inflammatory responses. Pretreatment of LPS led to an M1 polarization of macrophages that was positive for the iNOS, a typical surface marker for the proinflammatory M1 phenotype. However, further treatment with hydrogels induced the transformation of macrophages into spindle-shaped morphology and positive staining for M2 phenotype-specific markers CD206(Figure 4I). These results indicate the successful induction of M1 to M2 polarization of GT4MKS hydrogels and suggest their potential as anti-inflammatory agents.

The migration, retention, and appropriate differentiation of endogenous stem/progenitor cells have been demonstrated to play a pivotal role in the regeneration of AF. SDF-1α and KGN have been extensively reported as effective biological components in disc repair, indicating the potential of their combination for cartilage and disc regeneration 34, 35. However, due to its instability, SDF-1α as a cytokine is less desirable than the more stable SDF-1α mimic polypeptide. The peptide could provide similar bioactivity while at the same time being easier to synthesize and store. Additionally, hydrophobic KGN can be properly delivered and controlled through coating with TA and fabrication into KGN-loaded nanofibers, offering a novel drug delivery method for hydrophobic compounds with a simple fabrication process. As such, the Geln/TA/Mn^2+^/KGN@TA/SDF-1α hydrogel composition provides an AF tissue mimic structure and promotes differentiation of recruited stem/progenitor cells towards specific phenotypes.

To enhance the recruitment of endogenous MSCs to the defect area and expedite the regeneration process, we have confirmed the release behavior of SDF-1α, a well-established stem cell recruitment cytokine. The cell recruitment capacity of hydrogels was evaluated through a scratch test, wherein a straight scratch was made across the monolayer of BMSCs, and the wound healing rate was recorded at 12-h intervals. A remarkably improved scratch size recovery rate in the GT4MKS group indicated exceptional stem cell homing ability of the SDF-1α loaded hydrogel (Figure 4J and Figure S12). When hydrogels were placed in the lower chamber of the 24-well trans-well plate, BMSCs seeded in the upper chamber were attached to pass through the membrane. The GT4MKS group also recorded a higher cell number and cell viability (Figure S13).

Given the anticipated recruitment of endogenous MSCs to the defect area, we further validated the appropriate induced differentiation of homing MSCs in hydrogel samples using a trans-well system. KGN has been identified as a chondrogenic agent capable of promoting MSC chondrogenesis. The selective differentiation of BMSCs stimulated by hydrogel samples was confirmed through gene expression analysis of key chondrogenesis biomarkers, Sox9, aggrecan, and collagen type II, after 1- and 3-week treatment (Figure 4K). There was no significant trend of osteogenic differentiation, as evidenced by the low expression levels of the osteogenesis biomarkers RUNX2 (Figure S14A) and ALP (Figure S14B). Notably, treatment with GT4MK showed comparable chondrogenic effects to direct supplementation with KGN and even exhibited superior induction at week 3. Western blot analysis of collagen type II and aggrecan confirmed the chondrogenic differentiation of co-cultured BMSCs at the protein level (Figure S15A and S15B). Additionally, degradation metabolism biomarkers MMP13 and ADAMTS-5 were downregulated simultaneously (Figure S15C). All the evidence provided above demonstrates a solid foundation for designing new strategies that utilize physical cues of the scaffold to improve AF repair.

### Evaluation of AF regeneration *in vivo* with GTMKS gel

A 2 mm in diameter and 4 mm in depth cylindrical AF defect model was constructed on the anterolateral intervertebral discs in rabbits and treated with the implantation of corresponding hydrogels. Imaging tests, including X-ray and MRI, were conducted, and specimens were collected for further evaluation at 4- and 12-week post-operation.

The disc height index (DHI%) was recorded, and at 4 weeks post-surgery, the index in the GTM group was found to be comparable to that in the GTMK group but significantly higher than both the Blank and TA groups.

**Figure 5:**
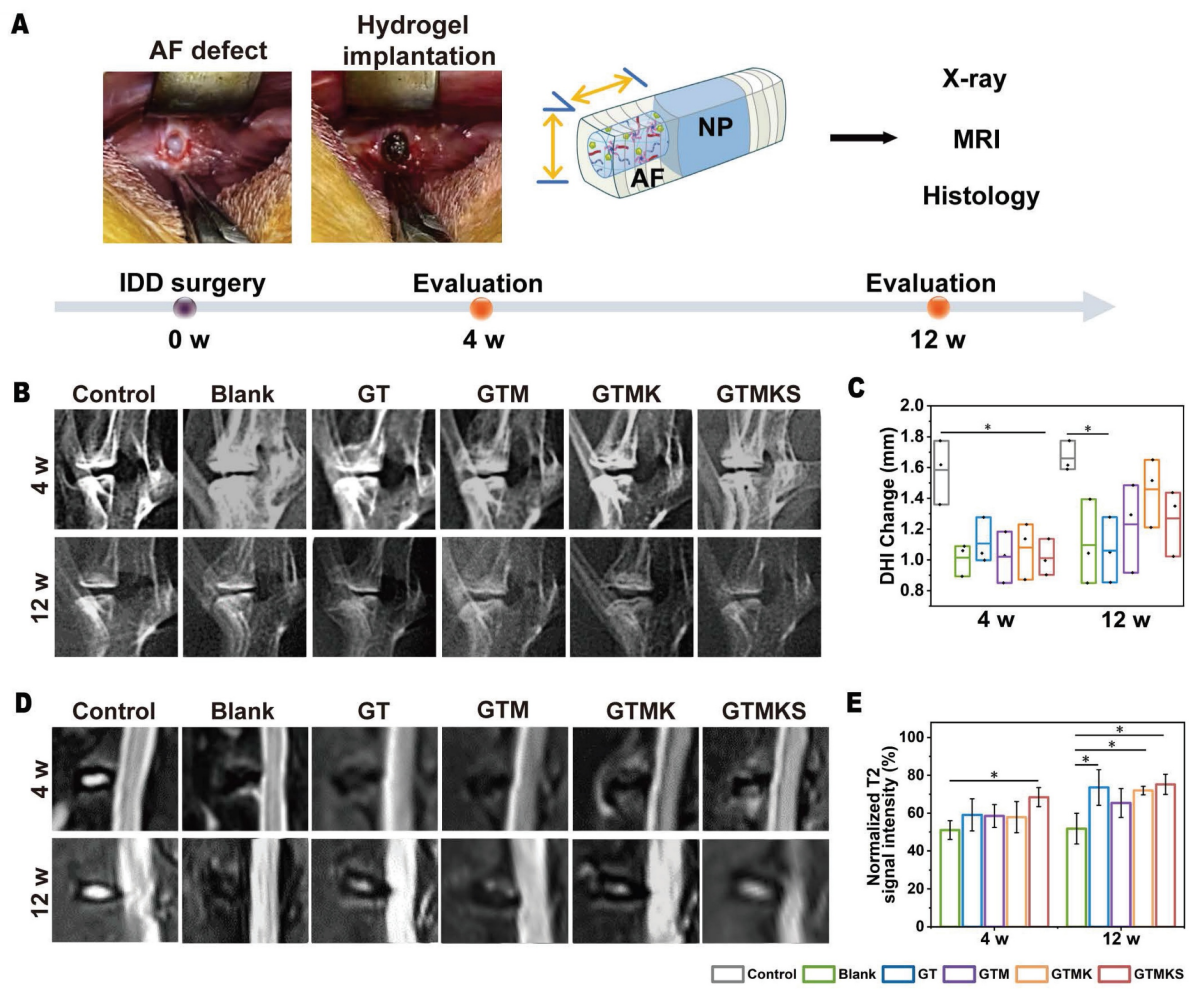
*In vivo* evaluation of IVD regeneration by GTMKS hydrogels. (A) Schematic illustration showing the overall procedure of the *in vivo* experiment. (B) X-ray images of rabbit lumbar vertebral discs in different groups at 4- and 12-week postoperative. (C) Quantitative DHI changes according to X-ray images at 4 weeks (n=6 discs) and 12 weeks (n=6 discs) postoperative. (D) MRI images and (E) normalized T2 signal intensity of IVDs in different groups at 4- and 12-weeks postoperative (n = 6 discs).

Notably, DHI% values in the GTMK group were similar to those in the control group, while a decrease in DHI% values was observed in the GTM and Blank group at 12 weeks post-surgery. Furthermore, there were no significant differences in DHI% values between the AF injured group and the TA crosslinked hydrogel group after both 4 and 12 weeks. Throughout the postoperative period, DHI% was significantly reduced in both GT and Blank groups compared with the normal control group (p < 0.05), whereas no significant difference was found between Blank and GTM groups (p < 0.05) (Figure 5B and 5C).

The assessment of water content in the nucleus pulposus and its correlation with IVD can be achieved through MRI analysis of T2-weighted signal images. As depicted in Figure 5D, the signal intensity observed in T2-weighted MRI images was higher in both GTMKS and GTM groups compared to the injury group and GT group at 4 and 12 weeks post-surgery. The result suggests an increase in water content within the nucleus pulposus of IVDs in these two groups, thereby facilitating the restoration of their structure and function. When combined with simulated peptide-induced stem cell recruitment along with KGN induction, the GTMKS group exhibited further enhancement of signal strength at 12 weeks. The normalized T2 signal intensity (Figure 5E) and subsequent quantitative MRI grading results (Figure S16) were consistent with those obtained from MRI images.

**Figure 6:**
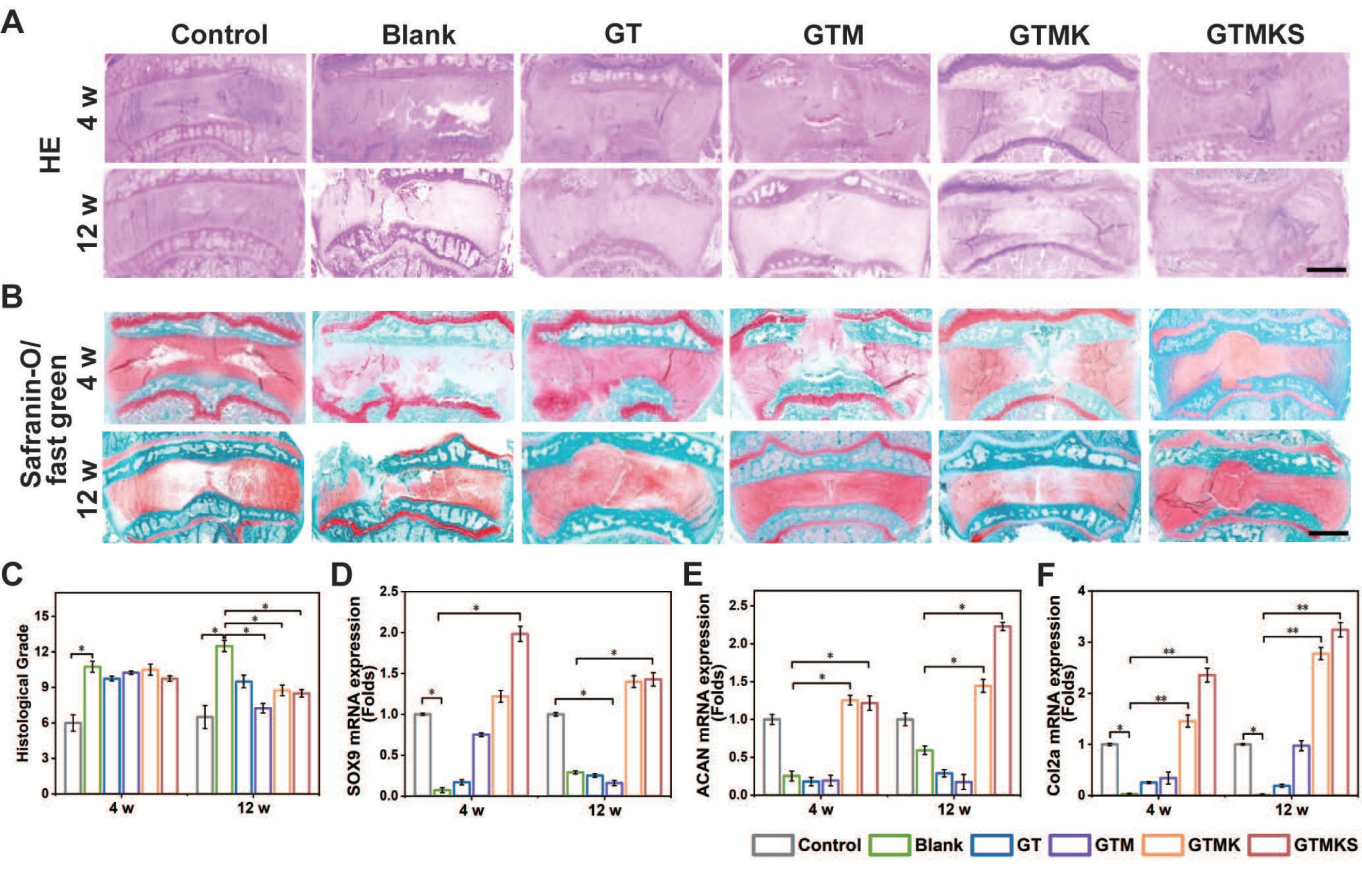
(A-B), H&E, safranin O, and fast green staining of IVDs in rabbits at 4 and 12 weeks postoperative (scale bar = 2.5 mm). (C), Changes in the histological grades at 4 and 8 weeks postoperative (n = 6). (D-F), gene expression level of SOX9, aggrecan and collagen type II with or without treatment (n = 6).

The histological evaluation was conducted to assess the regeneration of AF. As depicted in Figure 6A and 6B, H&E staining and safranin O/fast green staining revealed extensive infiltration of cells and a highly disorganized disc structure in both the injured group and hydrogel-implanted group at 4 and 12 weeks. The GTMKS group exhibited a slight reduction in NP areas at 4 weeks, which progressed to a more severe condition at 12 weeks. In contrast, the GTMK group demonstrated better-organized annulus fibrosus tissues compared to the other groups, except for the control group at 4 weeks, resembling the control group at 12 weeks.

Additionally, quantitative histological grading indicated lower scores in the co-delivery group compared to those observed in the injured, GT, and GTMKS groups after both time points (Figure 6C). Moreover, enhanced tissue-specific ECM expression was observed in the GTMK and GTMKS groups, particularly at 12 weeks post-treatment, as confirmed by gene expression levels of SOX9, aggrecan, and collagen type II. We performed an immunofluorescent analysis to further demonstrate the hydrogel system's remarkable regenerative efficacy.

The immunofluorescent staining results combined with gene expression data also indicated a downregulation of MMP13 in the drug-loaded hydrogel groups (Figure S17A and S17B), suggesting potential suppression of ECM catabolism. Notably, when compared to the sham group, significant suppression of Collagen type I expression was observed in the GTMKS group (Figure S17C), which may imply undesirable fibrous repair.

**Figure 7:**
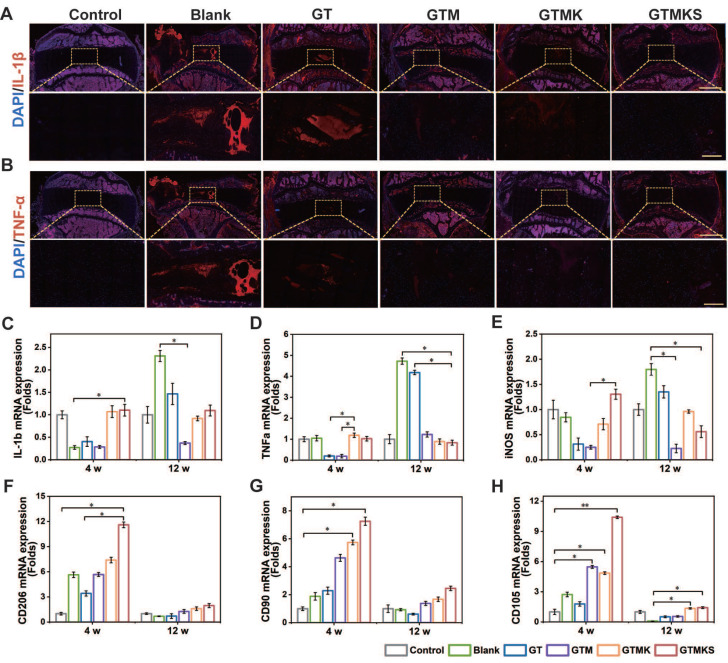
(A-B), Representative immunohistochemistry fluorescence of IL-1β and TNF-α in the disc (n = 6) at 12 weeks postoperative (scale bar = 2.5 mm in original Figure and 500 μm in magnified Figure). (C-H), Gene expression levels of IL-1β, TNF-α, iNOS, CD 206, CD90, and CD105 for the discs underwent different treatments at 4 and 12 weeks postoperative (n = 6).

After 12 weeks, samples stained with IL-1β and TNF-α were assessed for their potent anti-inflammatory capacity. Remarkably, the Mn2+-enriched TA/Geln hydrogel groups exhibited significantly reduced expression of inflammatory factors compared to the Blank group (Figure 7A and 7B). The gene expression levels of IL-1β and TNF-α were consistent with the immunofluorescent findings (Figure 7C and 7D). Additionally, at 12 weeks post-operation, a similar expression pattern was observed for IL-6 (Figure S18). Moreover, the early-stage upregulation of CD206 and subsequent downregulation of iNOS further support the anti-inflammatory properties of the dual drug-loaded hydrogel.

Overall, the tissue repair effect was observed to be superior after 12 weeks compared to 4 weeks within each group. Furthermore, the GTM group exhibited enhanced performance in promoting damaged AF repair when compared to the sham group, indicating that GTM alone could effectively facilitate AF repair. The GTMKS and GTMK groups demonstrated even better outcomes than the GTM group, with the GTMKS group exhibiting superior results over the GTMK group, potentially attributed to SDF-1α mimic peptide release, evidenced by the higher expression of stem/progenitor cell marker CD90 and CD105 (Figure 7G and 7H).

The introduction and sequential release of stem cell recruitment peptide and KGN significantly improved the AF repair effect of the hydrogel system, as evidenced by optimal performance observed in the GTMKS group. It is worth mentioning that the GTMKS complex played a crucial role in inhibiting pro-inflammatory cytokine expression and promoting the secretion of ECM-related cytokines. The evidence provided above confirmed that the GTMKS hydrogel effectively attenuated IVDD progression by alleviating inflammatory responses and regulating ECM metabolic balance within a specific microenvironment.

## Conclusion

Together, the present study may contribute to the development of a functionalized disc scaffold applied in an IDD environment toward annulus tissue engineering. An AF tissue-like Geln/TA/Mn^2+^/KGN@TA/SDF-1α composite hydrogel was proposed, aiming to induce a successful repair of the degenerated annulus fibrosus and enhance the intrinsic healing capabilities of the host tissue. The multifunctional polyphenol-based composite hydrogel demonstrates excellent biocompatibility, degradation, MSC recruitment, and ECM deposition properties, as well as remarkable antioxidant and anti-inflammatory properties. Moreover, it exhibited tunable mechanical strength, remolding, and self-healing properties, allowing it to fit in the narrow space within the IVDD without leakage. These findings highlight its potential as a promising candidate for biologically induced disc repair.

## Supplementary Material

Supplementary figures and tables.
