# Supplementary material for: Polyphenol and metal ion-reinforced supermolecular hydrogels incorporating nanofiber drug and peptide for annulus fibrosus regeneration
Source: Theranostics. 2025 Apr 21;15(12):5756–71. doi: 10.7150/thno.106913 (PMC12068310; doi:10.7150/thno.106913)
Supplement: Supplementary file 1 — Supplementary figures and tables. [file thnov15p5756s1.pdf]

Supplementary information

Polyphenol and metal ion-reinforced supermolecular hydrogels incorporating nanofiber drug and peptide for annulus fibrosus regeneration

Long Xin,[a,#] Xiaolin Li,[b,g,#,*] Yang Yang,[a] Pan Chen,[d] Yi Li,[e] Jianhua Liu,[f] Kangbo Chen,[c] Peipei Su,[c] Shuaishuai Feng,[c] Shiping He,[g] Xinwei Xu,[a] Wei Wang,[c,*] Weixing Xu,[a,*]

[a] Orthopedics Laboratory, Department of Orthopedics, Tongde Hospital of Zhejiang Province, Hangzhou, 310012, China.

[b] Engineering Research Center of Functional Materials Intelligent Manufacturing of Zhejiang Province, ZJU-Hangzhou Global Scientific and Technological Innovation Center, Zhejiang University, Hangzhou, 311215, China.

[c] College of Chemical and Biological Engineering, Zhejiang University, Hangzhou, 310027, China.

[d] Zhuji Affiliated Hospital of Wenzhou Medical University, Zhuji, 311899, China.

[e] The Second Affiliated Hospital of Zhejiang University, Hangzhou, 310009, China.

[f] College of Mechanical and Automotive Engineering, Zhejiang University of Water Resources and Electric Power, Hangzhou, 310018, China.

[g] Department of Clinical Laboratory, Shenzhen Second People's Hospital, Shenzhen, 518035, China.

[#] These authors contributed equally to the work.
[*] Corresponding author. E-mail address: xwxspine@163.com (Weixing Xu); wwgfz@zju.edu.cn (Wei Wang); irislxl@163.com (Xiaolin Li).

Materials and Methods

Materials

Gelatin, tannic acid, manganese chloride, kartogenin, collagenase type II, ethanol, 2,2-diphenyl-1-picryl hydrazyl (DPPH), 2,2'-Azinobis-(3-ethylbenzthiazoline-6-sulphonate) (ABTS),2-phenyl-4,4,5,5-tetramethylimidazoline-1-oxyl 3-oxide (PTIO), sodium hydroxide, bovine serum albumin (BSA), and hydrochloric acid were purchased from Sigma-Aldrich. SDF-1α mimic peptides were purchased from GenePharma. Dulbecco's Modified Eagle's Medium (DMEM), fetal bovine serum (FBS), trypsin, and penicillin/streptomycin were purchased from Invitrogen. PVDF membranes and 0.22 μm syringe filters were purchased from Millipore.

AF cells culture

Lumbar spine discs were harvested from six-month-old female New Zealand White rabbits (~2.5 kg) immediately postmortem. The AF tissue from discs was dissected and digested at 37 °C in DME/F-12 supplemented with 0.25% trypsin for 30 min, followed by centrifugation to collect the tissue. Subsequently, the collected tissue was then further digested in DME/F-12 containing 5% FBS and 300 units/mL collagenase for 12 h. Isolated cells were cultured in DME/F-12 with 10% FBS under 5% CO_2, and were prepared for experiments when reaching approximately 80% confluence.

Biocompatibility of hydrogels

The biocompatibility of hydrogels with rat BMSCs and rabbit AFCs was evaluated using a CCK-8 assay. A density of 3000 cells/well was directly seeded in 96-well plates, and the cell culture medium was replaced with or without hydrogel extractions. After every 24 h of incubation, each well was given 10 μL of CCK-8 solution. Following a 4-hour incubation at 37˚C, the absorbance at 450 nm was measured using a UV–Vis spectrophotometer. Cell viability was calculated using the following formula:

$$\text{Cell viability (\%)} = [A_{Sample} - A_{Blank}]/[A_{Control} - A_{Blank}] * 100\%$$

Where the absorbances of the working solution were designated as A_{Blandk}, while the group treated with or without hydrogel sample extractions were denoted as A_{Sample} and $A_{Control}$,

respectively.

Western blot assay

The protein expression of BMSCs was evaluated by western blot assay. The cells were treated with chondrogenesis culture medium with or without hydrogels for 3 weeks. Protein samples were collected with RIPA buffer, and the concentration of proteins was determined by the BCA kit. The 10% SDS-PAGE gels were used to separate the proteins, followed by a transfer process to collect proteins with PVDF membranes. After being immersed in 5% BSA at room temperature for 1h, the membranes were incubated separately with Rabbit anti-collagen type II (1:800, EPR27418-25, Abcam, USA), Rabbit anti-aggrecan (1:500, ab3773, Abcam, USA), Rabbit anti-ADAMTS5 (1:250, ab41037, Abcam, USA), Rabbit anti-MMP-13 (1:3000, ab39012, Abcam, USA), and Rabbit anti-β-actin (1:3000, ab8227, Abcam, USA) primary antibodies at 4 °C overnight. The membranes were then incubated with goat anti-rabbit IgG H&L (HRP) (1:3000, ab6721, Abcam, USA) secondary antibodies at room temperature for 2h. The protein bands were detected by the ChemiDoc MP Imaging System, and protein expressions were quantified by the ImageJ software.

Lap shear test

The lap shear test was conducted to quantitatively measure the adhesion strength of the GT4M hydrogels. A 10 × 10 × 1 mm hydrogel sample was placed at the end within two 30 × 10 mm substrates, such as wood, PP, glass, metal, and pork skin, with an overlap area of 10 × 10 mm. The lap shear tests were conducted at room temperature with a universal material testing machine. The adhesion strength of hydrogels with different substrates was measured, and each group was performed with at least 3 replicates.

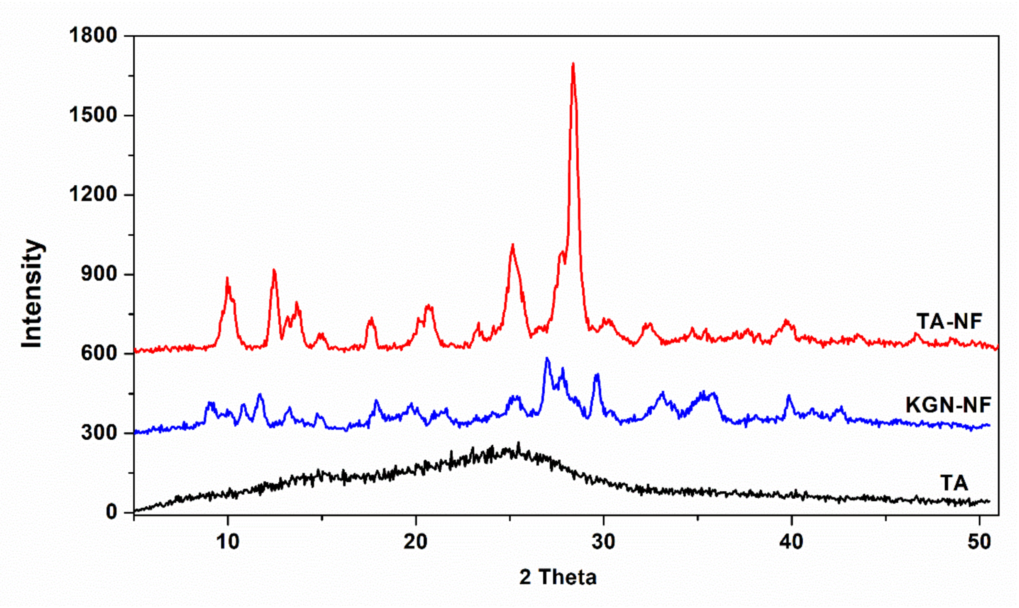

Figure S1. XRD figure of TA, TA nanofiber, and KGN@TA nanofiber.

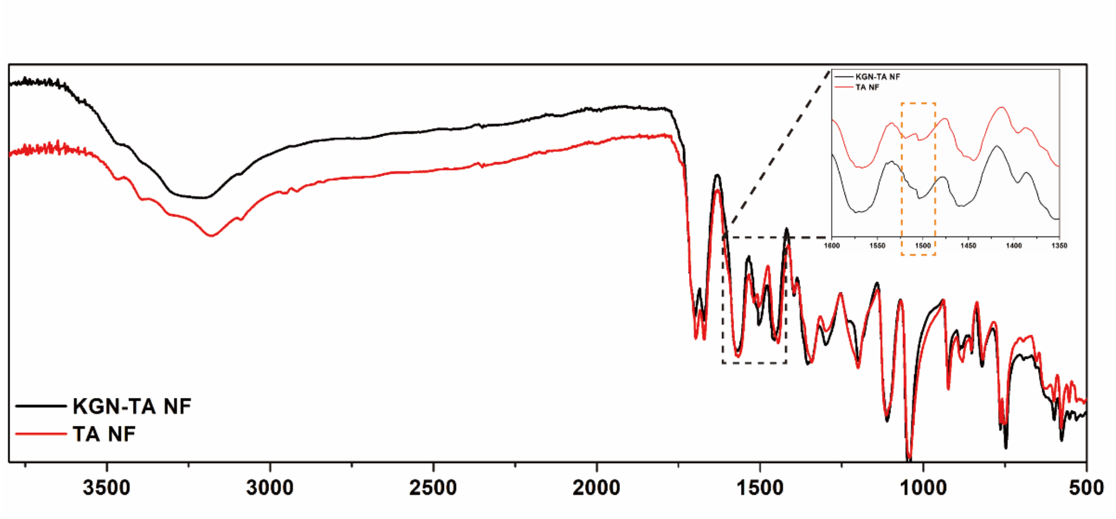

Figure S2. FTIR spectra of TA nanofiber and KGN@TA nanofiber.

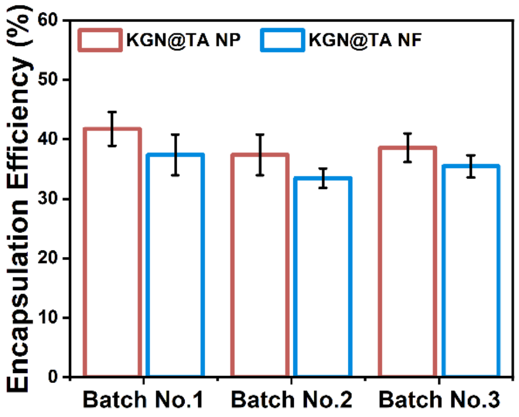

Figure S3. Encapsulation efficiency of KGN@TA nanofiber (n = 3).

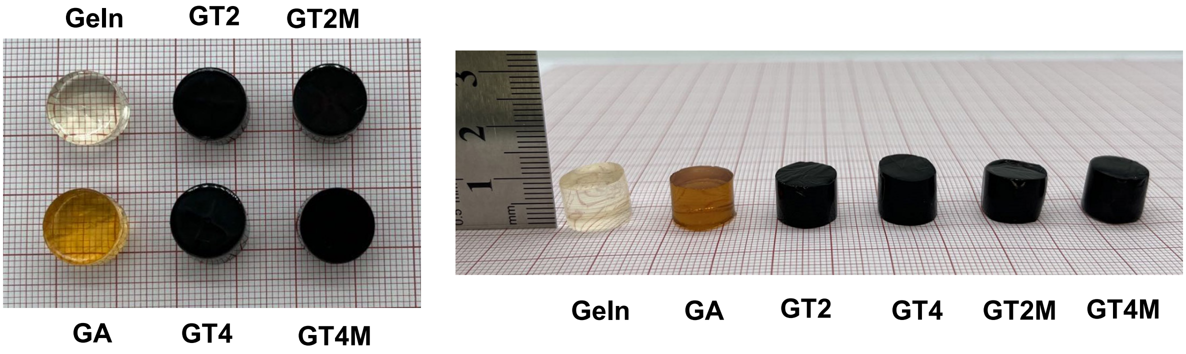

Figure S4. Macro image of hydrogels with various components.

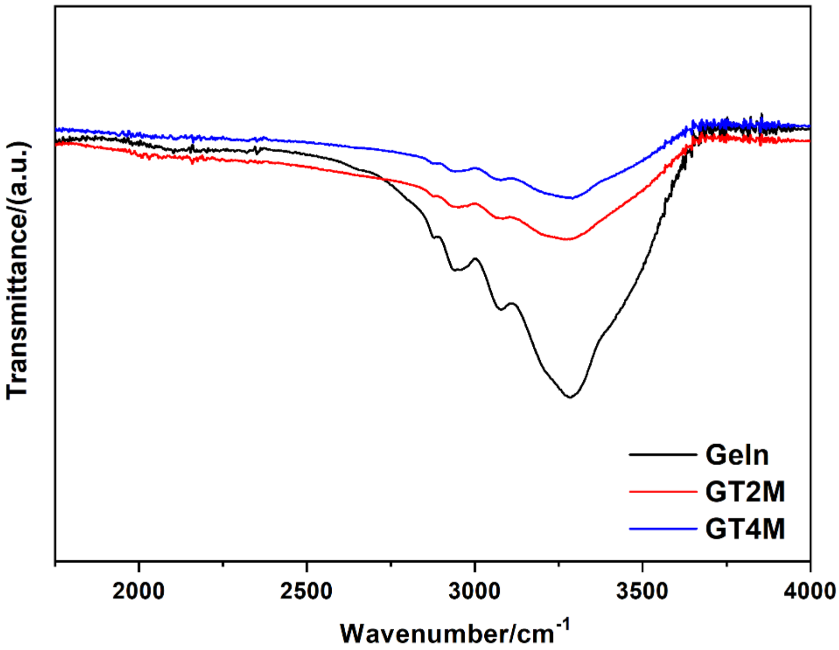

Figure S5. FTIR spectra of Geln, GT2M, and GT4M hydrogel.

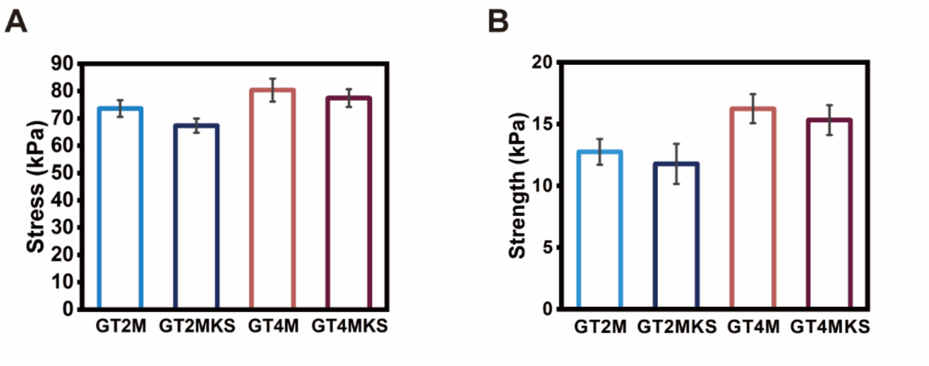

Figure S6. The summarised data of compressive stress under 50% applied strain (A) and stretch modulus (B) of different hydrogel samples (n=3).

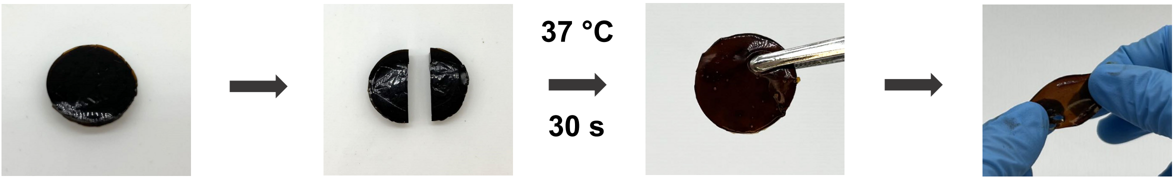

Figure S7. Images of GT4M hydrogel cut-recovery process.

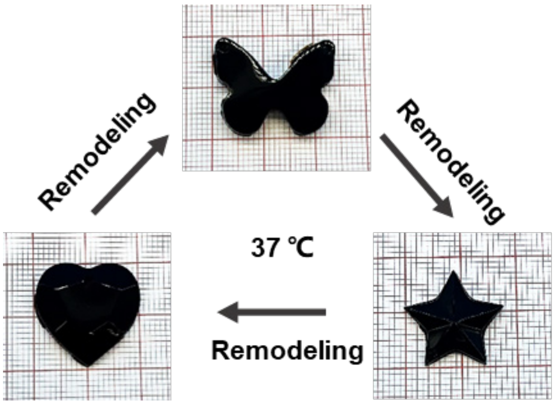

Figure S8. Images of GT4M hydrogel remodeling.

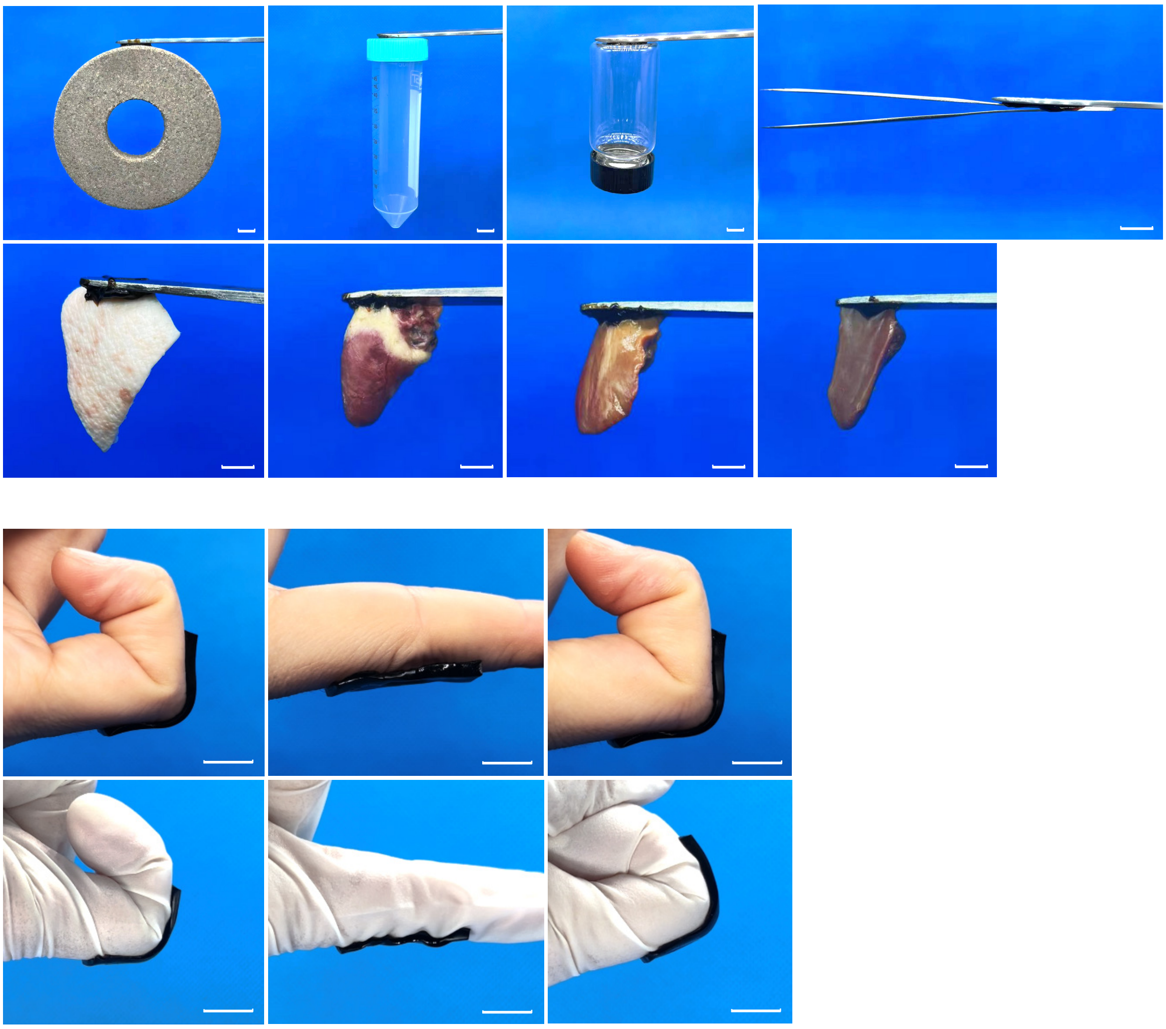

Figure S9. Adhesion behavior of GT4M hydrogels with various materials: wood, PP, glass, metal, pork skin, heart, lung, liver, skin, and latex. Scale bar = 1 cm.

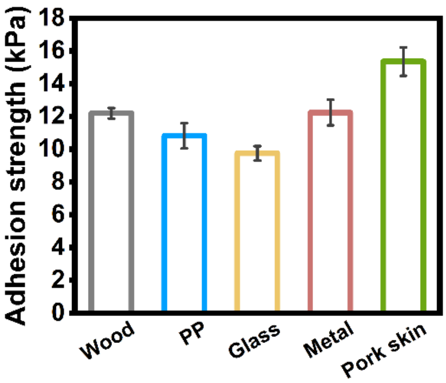

Figure S10. The adhesion strength of GT4M on wood, PP, glass, metal, and pork skin (n=3).

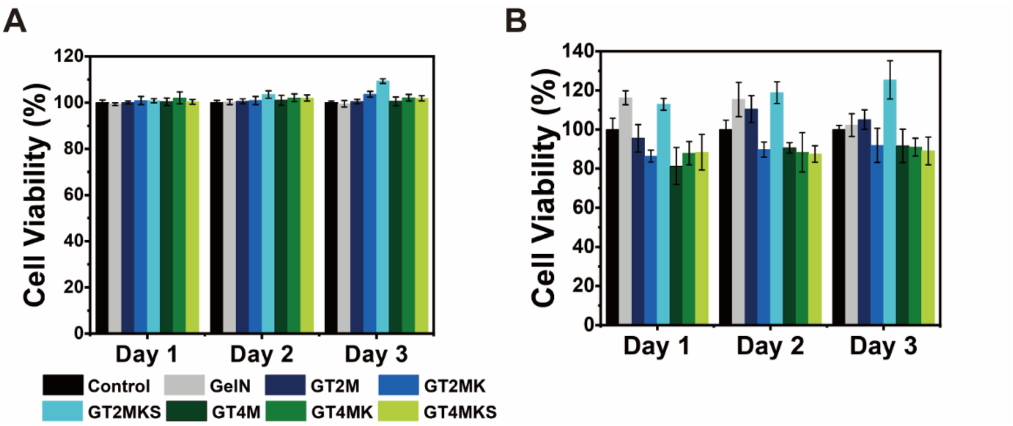

Figure S11. Cell viability of BMSCs (A) and AFCs (B) after culturing with hydrogels for 1, 2, and 3 days (n = 6).

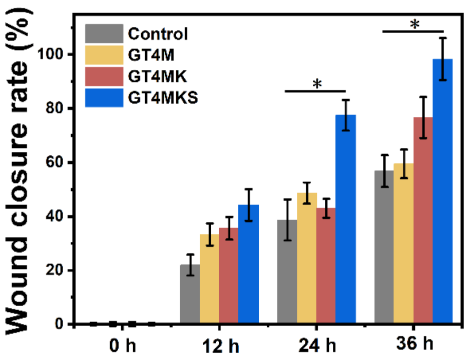

Figure S12. The wound closure rate of BMSCs with or without GT4M, GT4MK, and GT4MKS treatment.

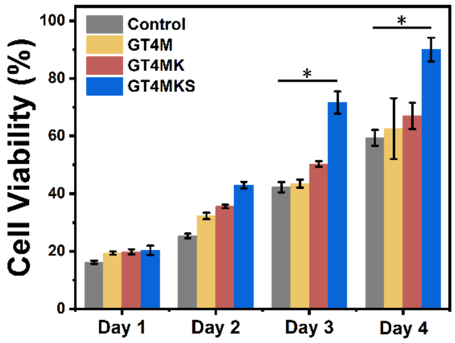

Figure S13. The cell viability of BMSCs with or without GT4M, GT4MK, and GT4MKS treatment.

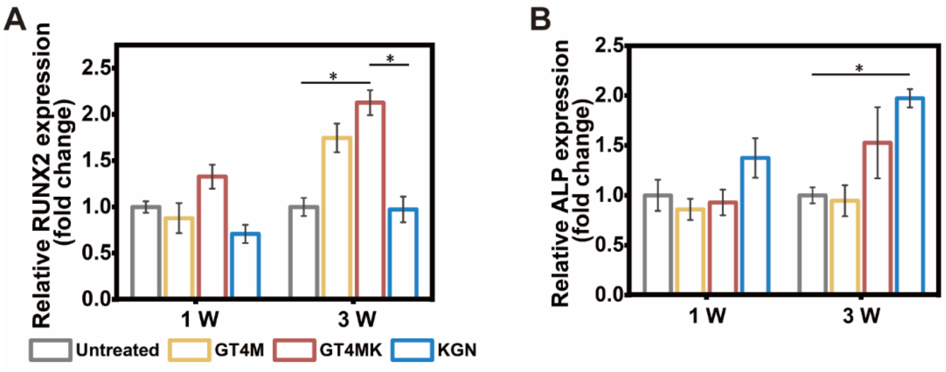

Figure S14. Gene expression of RUNX2 (A) and ALP (B) with or without GT4M, GT4MK, and KGN treatment in BMSCs.

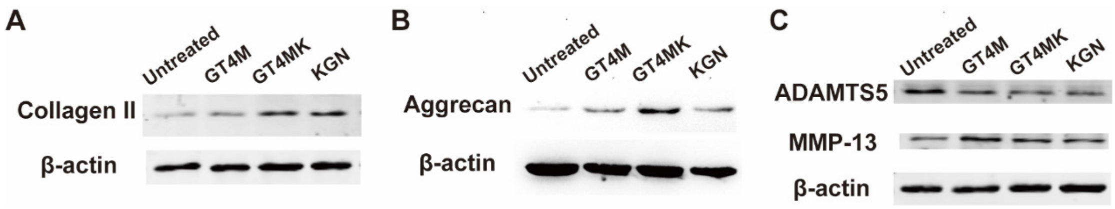

Fig S15. Protein expression of collagen type II (A), aggrecan (B), the MMP13, and ADAMTS5 (C) with or without GT4M, GT4MK, and KGN treatment in BMSCs

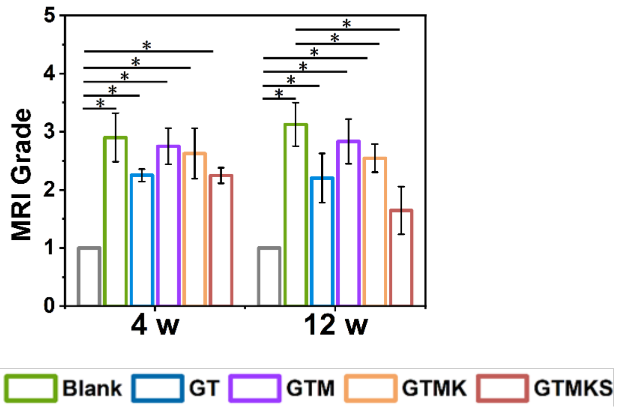

Figure S16. MRI grade of IVDs in different groups at 4 and 12 weeks postoperative (n = 3 discs).

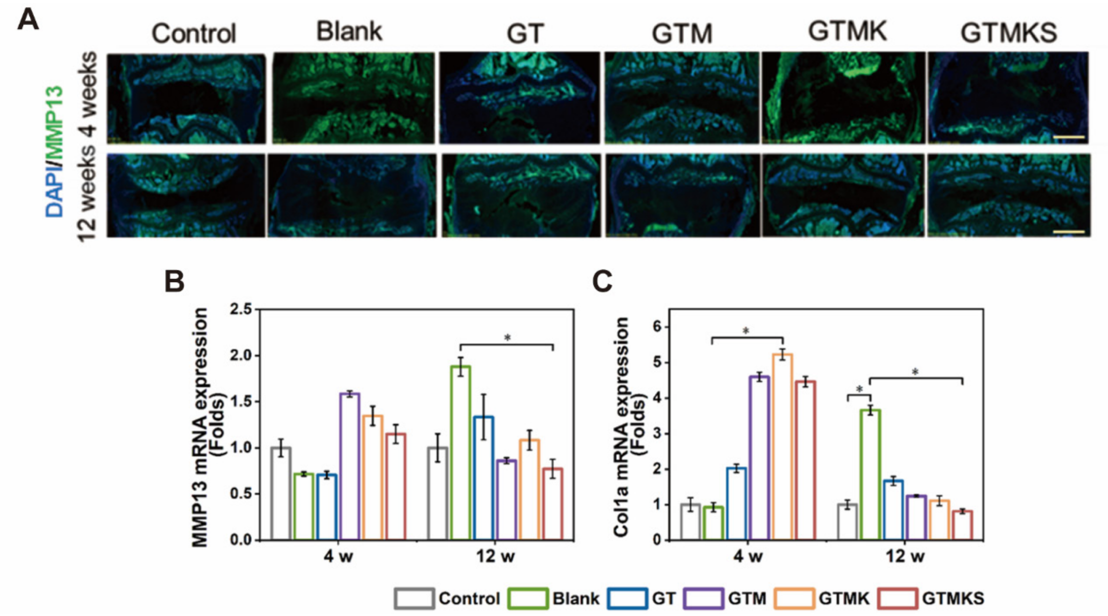

Figure S17. (A), Representative immunohistochemistry fluorescence of MMP-13 in the discs (n = 6) at 4 and 12 weeks postoperative (scale bar = 2.5 mm). Gene expression of MMP-13 (B) and Collagen type I (C) for the discs underwent different treatments at 4 and 12 weeks postoperative (n = 6).

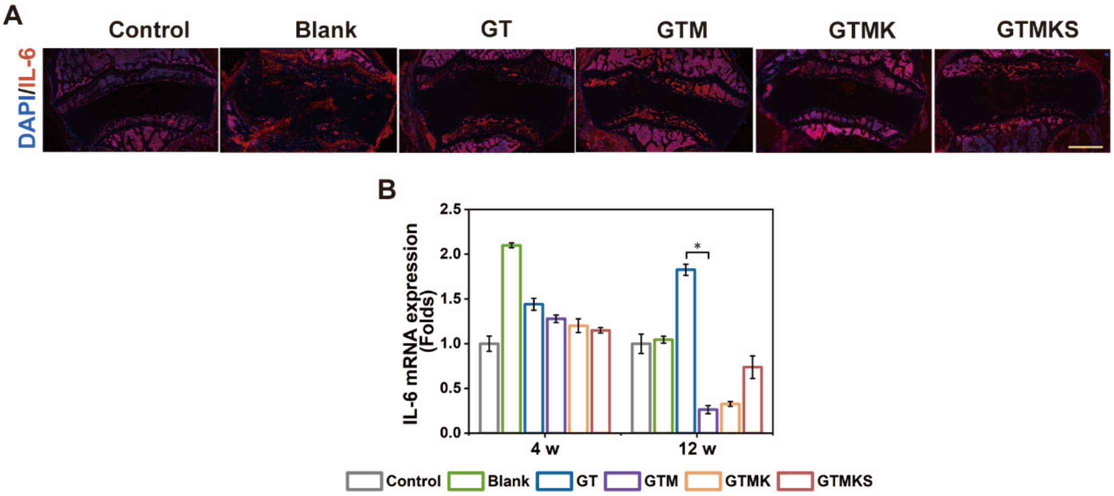

Figure S18. (A), Representative immunohistochemistry fluorescence of IL-6 in the discs (n = 6) at 12 weeks postoperative (scale bar = 2.5 mm). (B), Gene expression of IL-6 for the discs underwent different treatments at 4 and 12 weeks postoperative (n = 6).

Table S1. List of compositions of hydrogels prepared for *in vitro* experiments.

**Sample / Concentration**	**Gelatin (wt%)**	**TA (mg/mL)**	**$MnCl_2$ (mM)**	**Glutaraldehyde(wt%)**
**Geln**	**15**	**0**	**0**	**0**
**GA**	**15**	**0**	**0**	**1**
**GT2**	**15**	**20**	**0**	**0**
**GT4**	**15**	**40**	**0**	**0**
**GT2M**	**15**	**20**	**10**	**0**
**GT4M**	**15**	**40**	**10**	**0**

Table S2. Oligonucleotide primers for PCR amplification.

**Target name**	**Forward (5'-3')**	**Reverse (5'-3')**
Rat ACAN	CACAGGCAGCACAGACACTTC	GGAGTCAAGGTCGCCAGAGG
Rat Col2a1	GGCTCCCAGAACATCACCTA	GCCCTCATCTCCACATCATT
Rat SOX9	GCGAGCAGCAGCAGCACTC	TCTGGTGGTCGGTGTAGTCATACTG
Rat ALP	GCAACTTCCAGACCATTGGC	TCCCACTGACTTCCCTGCTT
Rat RUNX2	CGTGGCCTTCAAGGTGGTAG	GAGGCATTCCGGAGCTCAG
Rat β-actin	GGAGATTACTGCCCTGGCTCCTA	GACTCATCGTACTCCTGCTTGCTG
Rabbit Col2a1	CACGCTCAAGTCCCTCAACAAC	TCTATCCAGTAGTCACCGCTCTTC
Rabbit ACAN	GGAGGACTGCGTGGTGATGATC	AGGTGAAGGGTAGGTGGTAATTGC
Rabbit SOX9	GGCTCCGACACCGAGAATACAC	AACTTGTCCTCTTCGCTCTCCTTC
Rabbit Col1a2	GCAAGCGGCGGTGGTTAC	GTTCAGAGACTTCAGAGTGGCATC
Rabbit MMP13	TGAATCCTGCGGGAATCCTGAAG	CAAGTTTGCCTGTCACCTCTAAGC
Rabbit TNF-a	TGCTCCTCACTCACACTGTCAG	GACGCCGCCCAGGTAGATG
Rabbit IL-1b	TTGAAGAAGAACCCGTCCTCTG	GCCAGACAACACCAAGGATTTC
Rabbit IL-6	ACCTGCCTGCTGAGAATCAC	TCGTCACTCCTGAACTTGGC
Rabbit THY1(CD90)	CGCTGCTGCTGACAGTCTTG	AGGCTGAACTCGTGCTGGATG
Rabbit ENG(CD105)	GCATCAACACCAACCACAACATC	TGTCTGGGAGCGGGAAACC
Rabbit MRC1 (CD206)	TGGATGGCTCTGGTGTGGAAC	CTGGTAGGAAATGCTGGTCAATGG
Rabbit NOS2(iNOS)	CCTCAGAGTACAACAAGTGGAAGC	AGCAGGAAGGCAGCAGACAG
Rabbit GAPDH	CTGGTCATCAACGGGAAGGC	CTCCATGGTGGTGAAGACGC
